# Supplementary material for: Quantifying the deformability of malaria-infected red blood cells using deep learning trained on synthetic cells
Source: iScience. 2023 Nov 23;26(12):108542. doi: 10.1016/j.isci.2023.108542 (PMC10713842; doi:10.1016/j.isci.2023.108542)
Supplement: Document S1. Figure S1 [file mmc1.pdf]

iScience, Volume *26*

Supplemental information

Quantifying the deformability of malaria-infected red blood cells using deep learning trained on synthetic cells

Daniel T. Rademaker, Joshua J. Koopmans, Gwendolyn M.S.M. Thyen, Aigars Piruska, Wilhelm T.S. Huck, Gert Vriend, Peter A.C. `t Hoen, Taco W.A. Kooij, Martijn A. Huynen, and Nicholas I. Proellochs

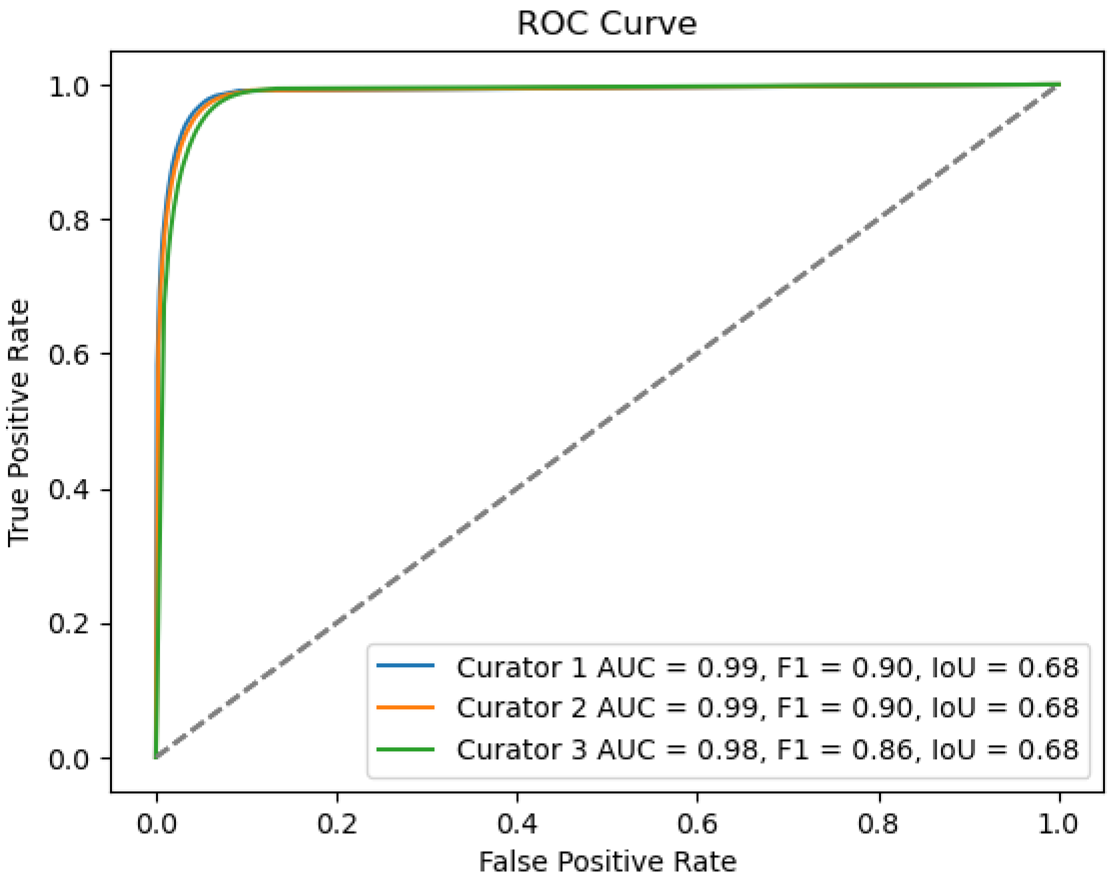

Figure S1: Comparison of Deep Learning segmentation with human curators, related to Table 1. The ROC curves presented in Figure S1 depict a comparison between the probability of the pixel predictions generated by Deep Learning segmentation with the labels provided by human curators. For each curator, the F1, AUC and IoU scores are shown.
